# HIF-1α Inhibition Reverses Multidrug Resistance in Colon Cancer Cells via Downregulation of MDR1/P-Glycoprotein

**DOI:** 10.1371/journal.pone.0098882

**Published:** 2014-06-05

**Authors:** Jianfang Chen, Zhenyu Ding, Yonghai Peng, Feng Pan, Jianjun Li, Lan Zou, Yanling Zhang, Houjie Liang

**Affiliations:** 1 Department of Oncology and Southwest Cancer Center, Southwest Hospital, Third Military Medical University, Chongqing, China; 2 Department of Oncology, General Hospital of Shenyang Military Region, Shenyang, Liaoning, China; Medical University of Gdańsk, Poland

## Abstract

**Background:**

Multidrug resistance (MDR) is one of the major reasons chemotherapy-based treatments fail. Hypoxia is generally associated with tumor chemoresistance. However, the correlation between the heterodimeric hypoxia-inducible factor-1 (HIF-1) and the multidrug resistance (MDR1) gene/transporter P-glycoprotein (P-gp) remains unclear. This study aims to explore the molecular mechanisms of reversing colon cancer MDR by focusing on the target gene HIF-1α.

**Methods:**

A chemotherapeutic sensitivity assay was used to observe the efficiency of MDR reversal in LoVo multicellular spheroids (MCS). The apoptotic level induced by different drugs was examined by flow cytometry (FCM). Binding of HIF-1α to the MDR1 gene promoter was evaluated by Chromatin immunoprecipitation (ChIP). The relationship between HIF-1α/P-gp expression and sensitivity to chemotherapy was analyzed.

**Results:**

The sensitivity of LoVo MCS to all four chemotherapy drugs was decreased to varying degrees under hypoxic conditions. After silencing the HIF-1α gene, the sensitivities of LoVo MCS to all four chemotherapy drugs were restored. The apoptotic levels that all the drugs induced were all decreased to various extents in the hypoxic group. After silencing HIF-1α, the apoptosis level induced by all four chemotherapy drugs increased. The expression of HIF-1α and P-gp was significantly enhanced in LoVo MCS after treatment with hypoxia. Inhibiting HIF-1α significantly decreased the expression of MDR1/P-gp mRNA or protein in both the LoVo monolayers and LoVo MCS. The ChIP assay showed that HIF-1α was bound to the MDR1 gene promoter. Advanced colon carcinoma patients with expression of both HIF-1α and P-gp were more resistant to chemotherapy than that with non expression.

**Conclusions:**

HIF-1α inhibition reverses multidrug resistance in colon cancer cells via downregulation of MDR1/P-gp. The expression of HIF-1α and MDR1/P-gp can be used as a predictive marker for chemotherapy resistance in colon cancer.

## Introduction

Colon cancer is one of the most common malignant tumors throughout the world, and chemotherapy plays an important role in its treatment. However, the sensitivity to different chemotherapeutic regimens varies widely from individual to individual. Many cancer patients develop drug resistance, leading to poor treatment outcomes. Moreover, drug resistance mostly occurs in solid tumors in vivo but not in monolayers in vitro [1]. The tumor microenvironment plays a pivotal role in chemotherapy failure and drug resistance [2]–[4].

Hypoxia is a common feature of many malignant tumors, including colon cancer. A hypoxic tumor microenvironment is increasingly considered a critical component in determining drug resistance [5], [6]. Hypoxia-inducible factor-1 (HIF-1) is a key factor in altering the biological characteristics of tumors. HIF-1α protein is overexpressed in multiple types of human cancer and is associated with worse prognosis in many cancers. Although many studies have indicated that hypoxia potentiates tumor resistance to chemotherapy and radiotherapy [7], [8], how the hypoxic microenvironment contributes to anticancer drug resistance has not yet been established.

Multidrug resistance (MDR) is one of the major reasons why chemotherapy-based treatment failure occurs. Of the many mechanisms of MDR, the high expression of the human MDR1 gene and the P-glycoprotein (P-gp) transporter encoded by MDR1 is an important focus of research [9]. Tumor cells that overexpress MDR1/P-gp usually show resistance to various chemotherapeutics [10], [11]. Our previous study showed that HIF-1α protein expression is correlated with MDR1/P-gp expression in colon carcinoma tissue and a colon cancer cell line, and mRNA expression levels of HIF-1α and MDR1 are significantly higher in the same type of cells in hypoxic conditions than in normoxic conditions [12]. It remains unclear whether and how HIF-1α is involved in MDR in colon cancer via the interaction of MDR1/P-gp.

Exploring the influence of hypoxia on colon cancer MDR will help improve the effect of chemotherapy. In hypoxic microenvironment, we assume that HIF-1α may directly induce the expression of MDR1/P-gp which leads to MDR, and the reversal of colon cancer MDR can be achieved when HIF-1α expression is specifically inhibited. In the present study, we aim to explore the potential molecular mechanisms and feasibility of reversing colon cancer MDR by focusing on the target gene HIF-1α. We expect to provide a theoretical basis and experimental evidence for later related applications in clinical treatment.

## Materials and Methods

### Cell Culture

The human colon cancer cell line LoVo was obtained from the American Type Culture Collection (Manassas, VA, USA) and cultured in high-glucose Dulbecco’s modified Eagle’s medium (DMEM, Gibco, USA), supplemented with 10% fetal bovine serum (HyClone, USA) and antibiotics (1% penicillin and 1% streptomycin). For hypoxia exposure, cells were cultured for 24 hours in a modulator incubator chamber at 37°C with 1% O_2_, 5% CO_2_, and 94% N_2_. Multicellular spheroids (MCS) were obtained by using the liquid overlay technique [13]. In brief, exponentially growing LoVo cells were added to culture medium plates that were previously coated with 2% agarose. The plates were gently and horizontally swirled for 10–15 minutes per 2–3 hours in the first 24 hours, and then for 10 minutes every 4 hours. Appropriate medium was refreshed every other day. For hypoxia experiments, appropriate MCS were established in normoxia for 48 hours and then cultured in hypoxia for another 24 hours.

### Chemicals

Adriamycin (ADR, Hisun Pharmaceutica, China), vincristine (VCR, Hisun Pharmaceutica, China), 5-fluorouracil (5-FU, Sigma, MO, USA), or irinotecan (CPT-11, Hengrui Medicine, China) were all freshly prepared on the day of use by dissolving the required concentration in DMEM with 10% fetal bovine serum.

### Chemotherapeutic Sensitivity Assay

LoVo cells were trypsinized, resuspended and counted, and 1000 cells were seeded into 96-well plates previously coated with 2% agarose to obtain MCS in normoxia. For hypoxia experiments, appropriate MSC were transferred into hypoxia for 24 hours and then incubated with gradient concentrations of drugs for another 24 hours in hypoxia. Cell viability was measured by the 3-(4, 5-dimethylthiazol-z-yl)-3, 5-diphenyltetrazolium bromide (MTT, BD Biosciences, NJ, USA) assay. Plates were incubated with MTT at 37°C for 4 hours, and the absorbance at 490 nm was measured by Model 550 Microplate Reader (Bio-Rad, CA, USA).

### Quantitative Real-time PCR

Total RNA was extracted with TRIZOL reagent (Invitrogen, CA, USA) according to the manufacturer’s instructions. The primers used were: 5′-GTTTGATTTTACTCATCCAT-3′ and 5′-TTCATAGTTCTTCCTCGG-3′ for HIF-1α; 5′-CTTGGCAGCAATTAGAAC-3′ and 5′-TCAGCAGGAAAGCAGCAC-3′ for MDR1; 5′-CCTGGATACCGCAGCTAGGA -3′ and 5′-GCGGCGCAATACGAATGCCCC -3′ for 18sRNA. The first-strand cDNA synthesis was performed with cDNA synthesis kit (TaKaRa, Dalian, China). Quantitative real-time PCR was performed using the SYBR Green real-time PCR kit (TaKaRa). All normalizations were done using 18sRNA levels and the fold changes were calculated by the delta–delta Ct method. All experiments were performed in three biological replicates.

### Western Blot Analysis

Total protein (50 µg) was separated by sodium dodecyl sulfate–polyacrylamide gel electrophoresis. After protein transfer to polyvinylidene fluoride microporous membranes (Bio-Rad), the membranes were blocked with 5% nonfat dry milk and incubated sequentially with the primary antibodies (HIF-1α 1∶500; P-gp 1∶200; β-actin 1∶5000), followed by incubation with the fluorescein-linked anti-mouse (anti-rabbit) IgG (1∶1000) and then incubation with anti-fluorescein alkaline phosphatase-conjugated antibody (1∶5000). All antibodies were bought from Santa Cruz, CA, USA. The immune complexes were detected with the enhanced chemiluminescence reagent (Pierce, USA). For quantification, signals were densitometrically normalized to β-actin by Quantity One image analysis software (Bio-Rad).

### HIF-1α siRNA Construction and Transfection

Pre-miRNA RNAi sequences for the target genes HIF-1α and MDR1 were designed and synthesized. After the two specific miRNA RNAi expression vectors with the reporter gene EmGFP targeting the human HIF-1α and MDR1 genes, respectively, were constructed and identified by restriction enzyme digestion and sequencing, the RNAi plasmids of HIF-1α and MDR1 were transfected into LoVo cells using the liposome Lipofectamine 2000 (Invitrogen, CA, USA). Stable positive clones were selected using blasticidin. The degrees of knockdown of HIF-1α and MDR1 were identified by PCR. The RNAi plasmids with better suppression effects were selected for subsequent construction of miRNA lentivirus expression clones. Two miRNA lentivirus expression clones, pLenti6/V5-GW/EmGFP-miR-HIF-1α and pLenti6/V5-GW/EmGFP-miR-MDR1, were constructed using Gateway recombination techniques (Invitrogen) and co-transfected into 293FT cells with the ViraPower packaging mix (Invitrogen). The titer was examined after infecting NIH/3T3 cells with virus supernatant. Then, LoVo cells were infected with the two lentiviral vector-mediated miRNA RNAi systems and stably selected by blasticidin.

### Chromatin Immunoprecipitation (ChIP)

Cells were plated into 100-mm-diameter dishes and, after 24 hours, incubated with 1% formaldehyde for 10 minutes at 37°C to cross-link proteins to DNA. The cross-linking reaction was quenched by the addition of one-tenth volume of 1.25 mol/L glycine. Cells were washed twice with ice-cold 1×PBS; resuspended in radioimmunoprecipitation assay buffer and kept on ice for 30 minutes. Then, cell lysates were sonicated on ice with a Hielscher UP200S ultrasound sonicator (Hielscher Ultrasonics GmbH, Germany) until the crosslinked chromatins were sheared to yield DNA fragments between 200 and 1000 bp. Supernatants were incubated with salmon sperm DNA/protein-50% agarose slurry to reduce non-specific background. Immunoprecipitation was then performed overnight at 4°C with 5 µg of anti-HIF-1α antibody (Santa Cruz, CA, USA). These supernatants were supplemented with 5 Mol/L NaCl and heated overnight at 65°C to reverse protein-DNA cross-links. The immunocomplexes were further treated with DNase-free and RNase-free proteinase K, and the DNA was purified by phenol/chloroform extraction and ethanol precipitation. PCR was performed with primers specific for region which contains the hypoxia responsive enhancer site (5′-GCGTG-3′) of the MDR1 promoter [14]. The primers used were as follows: 5′-GGAGCAGTCATCTGTGGTGA-3′ and 5′-CTCGAATGAGCTCAGGCTTC-3′. As reported previously [24], human vascular endothelial growth factor (VEGF, an established HIF-1 target gene) was used as a positive control and primers flanking the hypoxia responsive enhancer of the VEGF promoter were 5′-GCCTCTGTCTGCCCAGCTGC-3′ and 5′-GTGGAGCTGAGAACGGGAAGC-3′. Immunoprecipitation with non-specific IgG (Santa Cruz) was performed as negative control. A sample representing linear amplification of the total input DNA was used as input control.

### Detection of Apoptosis by Flow Cytometry (FCM)

Cells were prepared and treated as described above and then stained with allophycocyanin (APC)-conjugated annexin V and propidium iodide (PI) for 10 minutes at room temperature, according to the manufacturer’s instructions (Annexin V-APC/PI Apoptosis Detection Kit; Jingmei Biotech, China). The population of annexin V-negative viable and annexin V-positive apoptotic cells was evaluated by FCM. Data were collected in a FACSCalibur instrument (BD Biosciences) and analyzed using CellQuest software (BD Biosciences).

### Patients and Tumor Specimens

One hundred twenty patients with histologically confirmed advanced colon carcinoma and who underwent 5-FU-based chemotherapy at the Southwest Hospital, Third Military Medical University, Chongqing, China, between 2004 and 2008 were eligible for this study. This study was reviewed and approved by the Ethical and Protocol Review Committee of the Southwest Hospital, Third Military Medical University and all patients provided written consent form. The patients ranged in age from 30 to 79 years (mean age, 54 years); 73 were male, and 47 were female. Chemotherapy response was evaluated by CT scan or other radiographic means after two cycles of treatment, adopting the Response Evaluation Criteria in Solid Tumors Group criteria. Based on their chemotherapy response, patients were classified as responders or non-responders. Tissue specimens were fixed in 10% formalin, embedded in paraffin, and cut into 4-µm serial sections. The expression of HIF-1α and P-gp was examined by immunohistochemistry as previously described [12]. Clear brown-yellow staining restricted to the nuclei, cytoplasm, or cell membrane indicated positive expression of HIF-1α or P-gp. Positive expression was recorded as (+) and negative expression as (−).

### Statistical Analysis

All data are provided as the mean ± SD. The results were analyzed by the chi-square test, Student’s t test, and one-way analysis of variance (ANOVA). *p*<0.05 was considered significant. Statistical analyses were carried out using SPSS Version 13.0 for Windows (SPSS Inc., Chicago, IL, USA).

## Results

### LoVo MCS are more Resistant to Chemotherapy than Monolayers in Hypoxic Conditions

**Figure 1:**
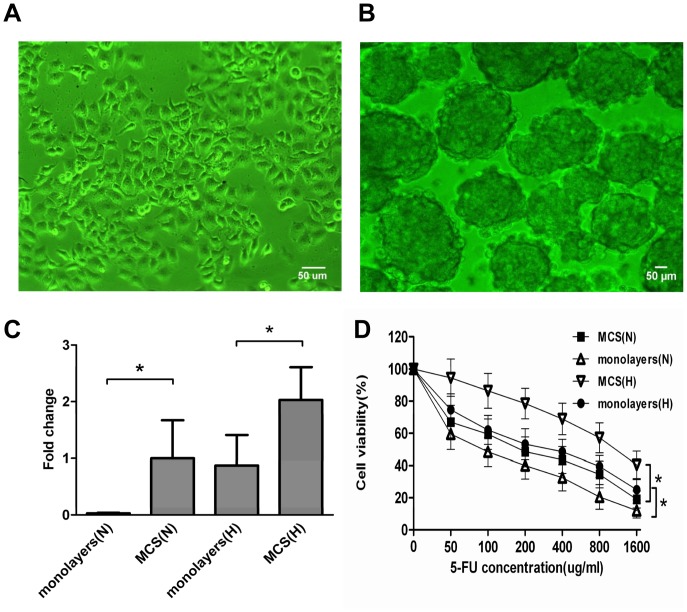
LoVo MCS show more resistance to chemotherapy than monolayers in hypoxia. (A) The morphology of LoVo monolayers. Scale bar = 50 µm. (B) The morphology of LoVo MCS. Scale bar = 50 µm. (C) Quantitative real-time PCR detected HIF-1α mRNA expression in LoVo MCS and in monolayers under normoxic and hypoxic conditions. The 18sRNA levels were used as internal control and the fold changes were calculated by the delta–delta Ct method. The experiments were performed in three biological replicates and PCR for each gene was done in duplicates (*p<0.05). (D) Drug sensitivities (mean ± SD, n = 3) of LoVo MCS and LoVo monolayers to 5-FU in hypoxia or normoxia (*p<0.05, N: normoxia, H: hypoxia).

To mimic the hypoxic microenvironment of a tumor in vivo [15], [16], LoVo MCS were obtained by using the liquid overlay technique. LoVo cells agglomerated with each other and proliferated rapidly. After being cultured for 48 hours, LoVo MCS were composed of a number of agglomerated cells (Figure 1B). Quantitative real-time PCR showed that the expression of HIF-1α was significantly higher in LoVo MCS than in LoVo monolayers, not only in hypoxic conditions but also in normoxia (*p*<0.05) (Figure 1C). Drug sensitivities to 5-FU were evaluated, and the results showed that LoVo MCS were more resistant to 5-FU than were monolayers in hypoxia (*p*<0.05). Moreover, both LoVo MCS and monolayers were more resistant to 5-FU in hypoxic conditions than in normoxia (Figure 1D).

### HIF-1α Inhibition Reverses Multidrug Resistance in LoVo MCS

**Figure 2:**
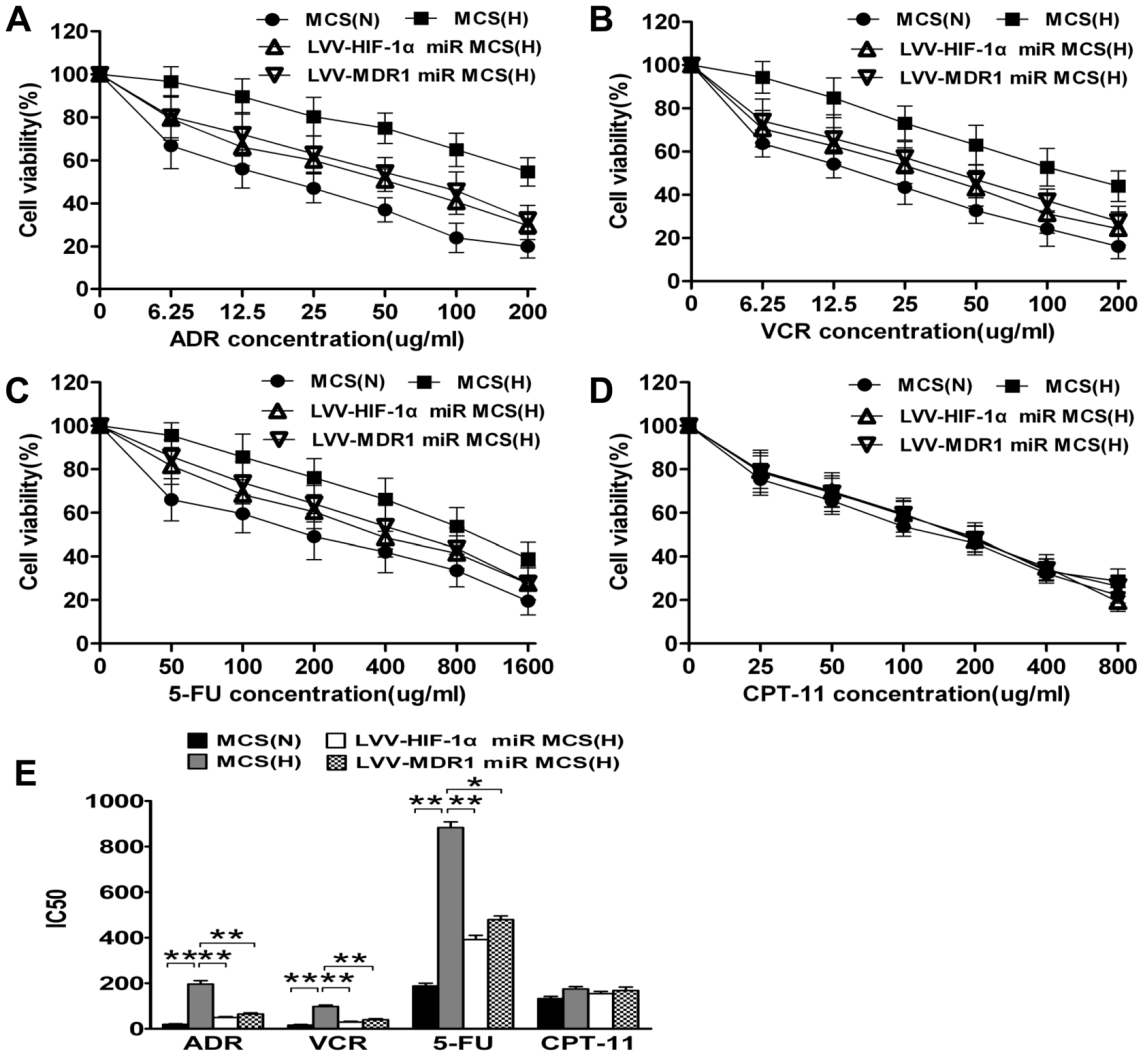
The sensitivity of different LoVo MCS groups to chemotherapy drugs was examined by MTT assay under normoxic and hypoxic conditions. (A) ADR. (B) VCR. (C) 5-FU. (D) CPT-11. (E) IC50 of different LoVo MCS groups to chemotherapeutics (mean ± SD, n = 3, **p<0.01, *p<0.05, N: normoxia, H: hypoxia).

Because LoVo MCS showed more resistance to chemotherapy than monolayers did in hypoxia, we wanted to investigate whether drug resistance changed when HIF-1α expression was specifically inhibited. We successfully established a LoVo MCS model and stable LVV-HIF-1α and LVV-MDR1 miR-infected groups. In hypoxic conditions, the sensitivity of LoVo MCS to chemotherapy with each of the four drugs decreased to varying degrees. The 50%-inhibiting concentration (IC_50_) values in the hypoxic group were all higher than those in the normoxic group (ADR, VCR, and 5-FU, *p*<0.01; CPT-11, *p*>0.05) (Figure 2). After silencing the HIF-1α gene, the sensitivities of LoVo MCS to all four chemotherapy drugs were restored to different extents. The relative reversal ratio between LVV-HIF-1α miR-infected group (hypoxia) and the MCS (hypoxia) for each drug was as follows: ADR, 82.8% (*p*<0.01); VCR, 83.8% (*p*<0.01); 5-FU, 70.7% (*p*<0.01); and CPT-11, 48.5% (*p*>0.05). After silencing the MDR1 gene, the relative reversal ratio between LVV-MDR1 miR-infected group (hypoxia) and the MCS (hypoxia) for each drug was as follows: ADR, 74.2% (*p*<0.01); VCR, 70.9% (*p*<0.01); 5-FU, 58.1% (*p*<0.05); and CPT-11, 15.2% (*p*>0.05).

### HIF-1α Inhibition Enhances the Apoptosis Induced by Chemotherapy Drugs in LoVo MCS

**Figure 3:**
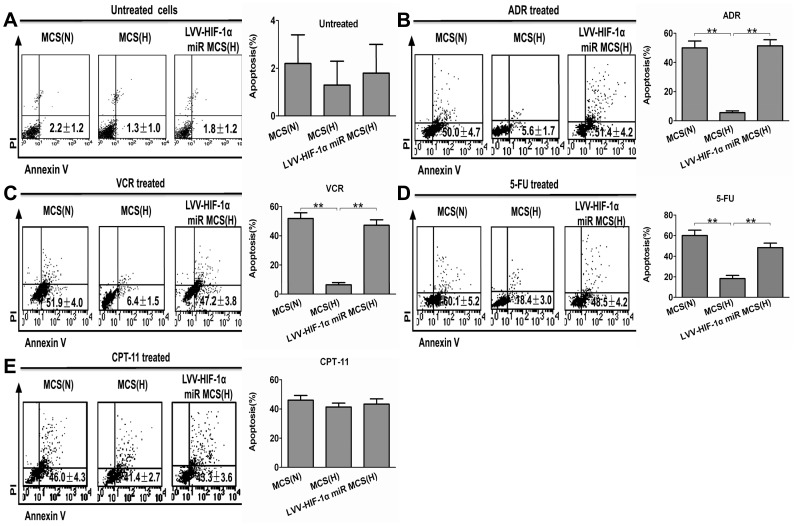
The change in the proportion of annexin V-positive apoptotic cells under normoxic and hypoxic conditions after treatment with chemotherapy drugs. The proportion of annexin V-positive apoptotic cells(lower right quadrant) was evaluated by flow cytometry using annexin V allophycocyanin (APC) and propidium iodide (PI) staining of LoVo MCS after treatment with (B) ADR (25 µg/ml), (C) VCR (25 µg/ml), (D) 5-FU (200 µg/ml) and (E) CPT-11 (200 µg/ml). Untreated cells were used as negative control. Data of each statistical graph of Annexin V-APC/PI staining is expressed in % of cells in the lower right quadrant and represent the mean ± SD of three independent experiments. The bar charts at the bottom of the flow cytometry scatterplots represent the % of cells undergoing apoptosis in the lower right quadrant (**p<0.01, N: normoxia, H: hypoxia).

We then observed the apoptosis induced by chemotherapy drugs in LoVo MCS when HIF-1α expression was specifically inhibited. The analysis of apoptosis by FCM demonstrated that (Figure 3), compared with LoVo MCS under normoxic conditions, the apoptotic levels induced by the four drugs were all lower to varying degrees in the hypoxic group (ADR, VCR, and 5-FU, *p*<0.01; CPT-11, *p*>0.05). After efficiently silencing the target gene HIF-1α, the induced apoptosis level by ADR, VCR and 5-Fu was remarkably increased (*p*<0.01) and was 9.2-fold, 7.4-fold, and 2.6-fold respectively in contrast to that of LoVo MCS (hypoxia group). Nevertheless, the apoptosis level induced by CPT-11 was only 1.1-fold to hypoxia group, with no statistical significance (*p*>0.05).

### MDR1/P-gp Protein Expression is Reduced in LoVo MCS after LVV-HIF-1α miR Transfection in Hypoxia

**Figure 4:**
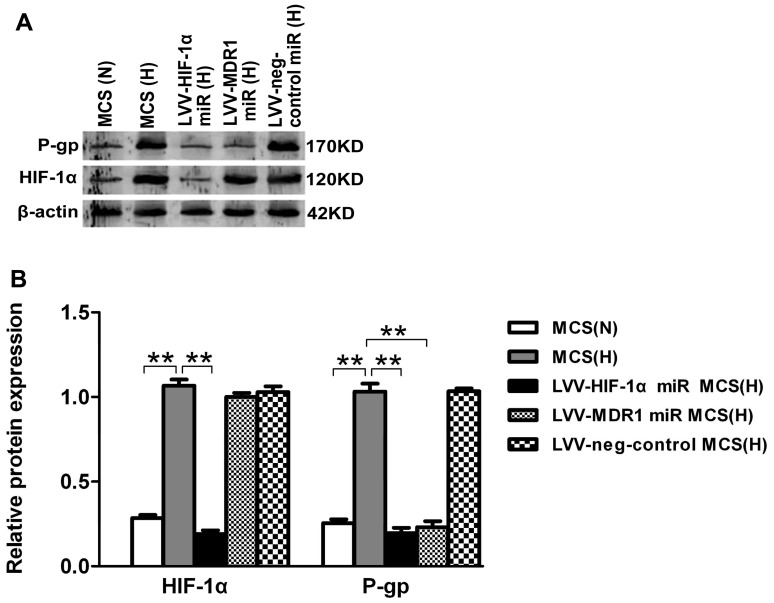
Expression levels of HIF-1α and P-gp in LoVo MCS with LVV-HIF-1α (or MDR1) miR stably transduced. (A) Western blot analyses were performed. β-actin was used as internal control. (B) Relative protein levels (mean ± SD, n = 3) of HIF-1α and P-gp were determined in each groups (**p<0.01, N: normoxia, H: hypoxia).

To explore the potential molecular mechanisms of reversing MDR by focusing on the target gene HIF-1α, we detected the expression of MDR-related genes in LoVo MCS when HIF-1α expression was specifically inhibited. Western blot showed that the expression of HIF-1α and P-gp in LoVo MCS was significantly enhanced after treatment for 24 hours in hypoxia (*p*<0.01). When HIF-1α was knocked down in the LVV-HIF-1α miR infected MCS, both HIF-1α and P-gp protein expression was downregulated compared with the negative control group (*p*<0.01). And, in the LVV-MDR1 miR infected MCS, the protein expression level of HIF-1α showed no difference while P-gp was reduced significantly compared with the negative control group (Figure 4). The results indicated that MDR1 was the downstream gene of HIF-1α and MDR1/P-gp expression will be reduced in LoVo MCS while HIF-1α was inhibited.

### MDR1/P-gp mRNA and Protein Expression are Reduced in LoVo Monolayers after LVV-HIF-1α miR Transfection in Hypoxia

**Figure 5:**
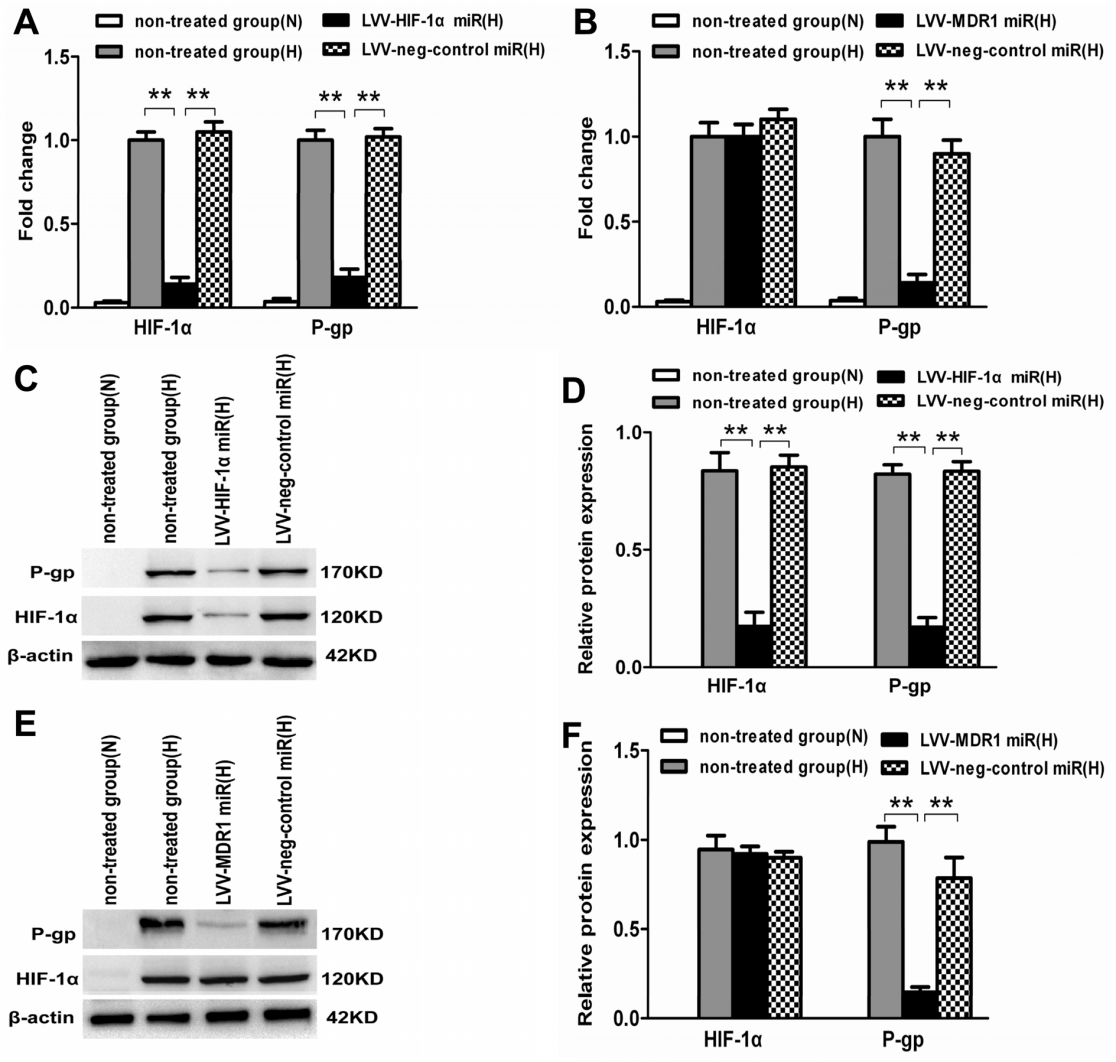
Expression levels of HIF-1α and MDR1/P-gp mRNA and protein in LoVo monolayers with LVV-HIF-1α miR stably transduced. (A, B) Quantitative real-time PCR detected HIF-1αor MDR1 mRNA expression in LoVo monolayers (hypoxia) with LVV-HIF-1α miR (A) or LVV-MDR1 miR (B) stably transduced. Non-treated monolayers in normoxia were used as normoxic control. The 18sRNA levels were used as internal control and the fold changes were calculated by the delta–delta Ct method. The experiments were performed in three biological replicates and PCR for each gene was done in duplicates (**p<0.01). (C–F) Western blot detected HIF-1α and P-gp protein expression in LoVo monolayers (hypoxia)with LVV-HIF-1α miR (C) or LVV-MDR1 miR (E) stably transduced. Non-treated monolayers in normoxia were used as normoxic control. (D, F) Comparison of expression levels (mean ± SD, n = 3) of HIF-1α and MDR1 protein of each group (**p<0.01).

Because MDR1/P-gp protein expression was reduced in LoVo MCS after LVV-HIF-1α miR transfection, we further detected the expression of MDR-related genes in the LoVo monolayers in hypoxia. After stably infecting LoVo monolayers with LVV-HIF-1α miR and LVV-MDR1 miR, Quantitative real-time PCR and Western blot (Figure 5) demonstrated that in hypoxia, the mRNA and protein expression levels of HIF-1α and MDR1 in the LVV-HIF-1α miR group were significantly lower than those of the non-treated and negative control miR groups (*p*<0.01). However, in the LVV-MDR1 miR group, only the mRNA and protein expression levels of the MDR1 gene were statistically lower than those of the non-treated and negative control miR groups in hypoxia (*p*<0.01), whereas no differences in HIF-1α mRNA and protein were noted (*p*>0.05).

### Binding of HIF-1α to the MDR1 Gene Promoter

**Figure 6:**
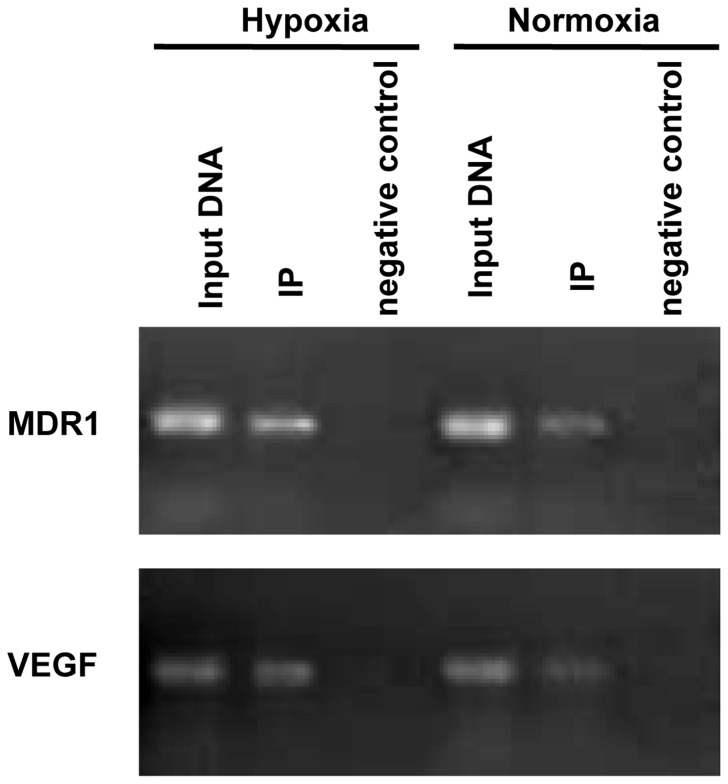
HIF-1α binds to the MDR1 and VEGF gene promoters in LoVo MCS. The binding of HIF-1α on the MDR1 and VEGF gene promoters was measured by ChIP. PCR analysis for the MDR1 gene promoter region was performed on immunoprecipitation samples (IP) with anti-HIF-1α antibody and with purified total input DNA from the LoVo MCS (normoxia and hypoxia). The VEGF gene promoter (an established HIF-1 target gene) was used as a positive control. Immunoprecipitation with non-specific IgG was performed as negative control. A sample representing linear amplification of the total input DNA was used as input control.

We then utilized a ChIP assay to evaluate in LoVo MCS (both in normoxia and hypoxia) whether HIF-1α bound to the promoter region of the MDR1 gene. After precipitation of cell lysates with a specific anti-HIF-1α antibody, the MDR1 gene promoter and VEGF gene promoter (an established HIF-1 target gene) were amplified by PCR with specific primers. The results (Figure 6) showed that HIF-1α was bound to the MDR1 gene promoter in LoVo MCS not only in hypoxic conditions but also in normoxic conditions.

### Patients with HIF-1α (+)/P-gp (+) are more Resistant to Chemotherapy

Our previous study has shown that HIF-1α and MDR1/P-gp expression levels correlate in tumor tissues of colon carcinoma [12]. To determine whether the expression of HIF-1α and MDR1/P-gp in colon cancer patients differs, as well as the related sensitivity to chemotherapy, immunohistochemical techniques were employed to detect the expression of HIF-1α and P-gp in the tumor tissues of 120 patients with advanced colon carcinoma who received 5-FU-based chemotherapy. We found that 73 of 120 cases (60.8%) expressed both HIF-1α and P-gp, and this HIF-1α (+)/P-gp (+) patient population was more resistant to chemotherapy than was the HIF-1α (−)/P-gp (−) population (*p* = 0.001, OR 5.647, 95% CI 1.920–16.606) (Table 1).

**Table 1:** The correlation between HIF-1α and P-gp expression and chemotherapy sensitivity.

	N	non-responders N (%)	Responders N (%)	*p* value*
HIF-1α (−)/P-gp (−)	19	7 (36.8)	12 (63.2)	
HIF-1α (+)/P-gp (−)	20	11 (45.0)	9 (55.0)	0.256
HIF-1α (+)/P-gp (+)	73	56 (76.7)	17 (23.3)	0.001
HIF-1α (−)/P-gp (+)	8	4 (50.0)	4 (50.0)	0.675

N: Number of patients;

*chi-square test; positive expression was recorded as (+) and negative expression as (−).

## Discussion

Our previous study showed that HIF-1α protein expression is correlated with P-gp expression in colon carcinoma tissues and colon cancer cell lines, and HIF-1α and MDR1 mRNAs were found to be significantly higher in the same cells under hypoxic conditions than under normoxic conditions [12]. In the present study, we show that HIF-1α may directly influence the expression of MDR1/P-gp in hypoxic microenvironment and then mediate chemotherapy resistance not only in cultured cell monolayers but also in multicellular spheroids, and HIF-1α inhibition may reverse multidrug resistance via downregulation of MDR1/P-gp. Much evidence has demonstrated that a hypoxic microenvironment is conducive to the development and maintenance of cancers [17]. Moreover, cancer cells in hypoxic conditions show more resistance to antineoplastic drugs [18], [19]. Some studies that have tried to explain this phenomenon have found that cells in hypoxic conditions generally divide slower than those in normoxic conditions, rendering therapies that target rapidly growing cells less effective [17]. Meanwhile, a study demonstrated that hypoxia could induce a number of genes, including MDR1/P-gp, in multicellular spheroids [16]. In our study, we found that HIF-1α bound to the MDR1 gene promoter in LoVo MCS not only in hypoxic conditions but also in normoxic conditions.

We successfully established a LoVo MCS model to mimic the hypoxic microenvironment of tumors in vivo. The chemotherapy sensitivity and apoptotic changes of LoVo MCS to ADR, VCR, 5-FU, and CPT-11 were examined in normoxia and hypoxia. The results showed that the chemotherapy sensitivities and apoptosis of LoVo MCS in response to the four drugs were all decreased to different degrees in hypoxic conditions. Of the four cytotoxic drugs, 5-FU and CPT-11 are the most commonly used in colon cancer, whereas resistance to ADR and VCR is thought to be closely related to MDR1/P-gp [20]. Silencing the HIF-1α gene restored the sensitivities of LoVo MCS to ADR, VCR, and 5-FU and induced a marked increase in the apoptotic level of each corresponding drug (*P*<0.01). These results indicate that drug resistance of LoVo MCS can be reversed with a combination of the LVV-HIF-1α miR system and the above drugs. This finding concurs with the previous findings that inhibiting HIF-1α can enhance the sensitivity to chemotherapeutic agents in cancer cells such as fibrosarcoma, gastric cancer, and breast carcinoma [21]–[23]. Since Unruh *et* *al.* [24] found that the antiproliferative efficacy of carboplatin and etoposide is significantly enhanced by the inactivation of HIF-1α in mouse embryonic fibroblasts, the contribution of HIF-1α to drug resistance has been observed in various types of neoplastic cells in recent years [21], [25]–[29]. However, the available data on the relationship between HIF-1α and the chemoresistance of cancer cells are conflicting. For example, some studies have demonstrated that the inactivation of HIF-1α in normoxia has no effect on drug responses in neuroblastoma and lung adenocarcinoma cells [30], [31]. Recent studies have also argued for a resistance-mediating effect of HIF-1α, at least in some human cancers [4]. Additionally, we found there was a trend toward more sensitivity after HIF-1α or MDR1 inhibition, but the chemotherapy sensitivities to CPT-11 were not significantly different in the LoVo MCS groups. The result indicated that the mechanism of chemoresistance to CPT-11 did not depend on P-gp but rather went through another pathway, such as the CPT-11 metabolic product SN-38 [32]–[34].

The mechanism by which HIF-1α contributes to MDR is complex and remained unclear. HIF-1α may mediate MDR by regulating drug efflux, altering cell proliferation and survival, inhibiting DNA damage, or reprogramming metabolism. As one of the main members of the ABC transporter family, MDR1/P-gp reduces the intracellular concentrations of cytotoxic drugs by actively pumping the drugs out of cells and thereby protecting cancer cells from their antitumor effects [35]. MDR1 is a target gene of HIF-1. In recent years, the contribution of HIF-1-mediated P-gp expression to hypoxia-induced drug resistance has been observed in many tumor cells, such as gastric cancer, gliomas, and breast carcinoma [14], [21], [26], [36]. In this study, we showed that the expression of HIF-1α and P-gp was significantly enhanced in LoVo MCS after treatment in hypoxia. HIF-1α inhibition by transfecting cells with a specific siRNA for HIF-1α significantly decreased the expression of MDR1/P-gp mRNA and protein in both LoVo monolayers and LoVo MCS. Furthermore, HIF-1α was bound to the MDR1 gene promoter directly. We indicated that HIF-1α activation may directly induce the expression of MDR1/P-gp and then lead to MDR.

To determine whether the expression of HIF-1α and MDR1/P-gp in colon cancer patients differs, as well as the related sensitivity to chemotherapy, immunohistochemistry were employed to detect the expression of HIF-1α and P-gp in the tumor tissues of 120 patients with advanced colon carcinoma who received 5-FU-based chemotherapy. We found that 73 of 120 cases (60.8%) expressed both HIF-1α and P-gp, and this HIF-1α (+)/P-gp (+) patient population was more resistant to chemotherapy than was the HIF-1α (−)/P-gp (−) population. Therefore, HIF-1α/MDR1 may be a promising target in the colon cancer treatment, and the reversal of colon cancer MDR can be achieved when HIF-1α/MDR1 expression is specifically inhibited. We suggest that the expression of HIF-1α and MDR1/P-gp can be used as a predictive marker for chemotherapy resistance in colon cancer.

In conclusion, we report that HIF-1α inhibition reverses MDR in colon cancer cells, which is associated with downregulation of MDR1/P-gp. Our results may have broad clinical implications for colon patients with HIF-1α (+)/P-gp (+) expression patterns, and combination therapies aimed at modulating HIF-1α expression in concert with standard chemotherapy regimens may provide a strategy to overcome tumor resistance.
